# What constitutes the best sex life for gay and bisexual men? Implications for HIV prevention

**DOI:** 10.1186/1471-2458-13-1083

**Published:** 2013-11-20

**Authors:** Adam Bourne, Gary Hammond, Ford Hickson, David Reid, Axel J Schmidt, Peter Weatherburn, The EMIS Network

**Affiliations:** 1 Sigma Research, Department of Social & Environmental Health Research, London School of Hygiene & Tropical Medicine, London, UK

**Keywords:** Sexual health, HIV prevention, Gay and bisexual men, Best sex life

## Abstract

**Background:**

While a large body of research has sought to understand HIV transmission risk behaviours among gay men, bisexual men and other men who have sex with men (MSM), less attention has been paid to the wider sexual health and well-being of this population. While some community-based organisations aim to support a more holistic sense of sexual well-being there is little evidence to draw on to inform their interventions. The current study sought to explore gay and bisexual men’s conceptions of what constitutes the ‘best sex’.

**Method:**

The EMIS survey of 2010 recruited more than 180,000 respondents from 38 European countries to complete an online questionnaire about sexual health and behaviour. The 12,942 English language, UK-based responses to the open ended question, “*What’s your idea of the best sex life?”* were subjected to a detailed content analysis. A framework was devised to reflect and describe the key themes emerging from the data, which was then used to code all responses to one (or more) of these themes. Further statistical analysis sought to establish if and how responses differed according to key demographic variables.

**Results:**

Eight themes emerged that capture the diversity of gay and bisexual men’s sexual desires. Most common among responses was a desire for sex within committed relationships, followed by a desire for sex which is emotionally or psychologically connected. Men also expressed a desire for volume and variety in their sexual lives, and for sex that is free from physical, social or psychological harm. Comparative analysis identified that older men were less likely to idealise a relationship or emotional connection, but were more likely to specify the sexual acts or behaviours they wished to engage in.

**Conclusions:**

Attending to what men value or aspire to can help ensure interventions are engaging and meaningful to the target population. HIV prevention interventions need to attend to the broad range of sexual desires held by gay and bisexual men in delivery of holistic sexual health promotion that can help them to have the best sex with the least harm.

## Background

Gay men, bisexual men and other men who have sex with men (MSM) remain the group at highest risk for contracting HIV in the UK [1], as in many other parts of the world [2,3]. Given this obvious public health priority, a significant number of studies have sought to understand specific sexual risk behaviours [4-6], predictive factors for transmission or acquisition of the virus [7-9], risk management techniques [10,11], and HIV prevention needs [12,13]. With emerging understanding of how HIV treatments can limit onward transmission [14,15], a growing body of literature has also explored how MSM perceive and utilise these new HIV prevention options [16,17].

While research to better understand HIV risk behaviours has been widespread, comparatively little attention has been paid to the broader sexual health and well-being of MSM. Where non risk-related research has been conducted it has tended to focus on exploring sexual dysfunction (including premature or delayed ejaculation, erectile difficulties or libido) [18,19], satisfaction with monogamous or polygamous relationships [20,21] or pleasure associated with specific sexual acts, such as unprotected anal intercourse [22]. A notable exception is the Pleasure and Sexual Health study [23] in Australia, which reports on how gay men weigh up their desire to avoid acquiring or transmitting HIV with a strong desire to have sex that is pleasurable.

Recently published data has explored sexual (un)happiness among MSM at a broader level, including a study of sexual problems experienced by gay and bisexual men with diagnosed HIV in the UK [24], which identified that 70.5% of 1119 men had experienced one or more problems with sex within the previous 12 months. Most commonly these included a loss of libido, poor self-image or low self-esteem, too little or no sex or worries about passing HIV to sexual partners. The multi-national European Men who have sex with men Internet Survey (EMIS) of over 180,00 men reported that 36.8% of respondents were not happy with their sex life [25], rising to 53.0% of those who were not ‘out’ to any of their friends, family or colleagues about their attraction to men.

The extent of sexual unhappiness is a significant cause for concern, particularly among those organisations adopting a health promotion approach to preventing the onward transmission of HIV. Many charities and other community based HIV organisations across the world have drawn on the broad principles of the Ottawa and Bangkok charters [26,27] in the development of their institutional policies or strategic plans that seek to facilitate a sense of sexual well-being that extends beyond the absence of disease (see, for example: ACON in New South Wales, Australia [28]; Gay Men’s Health Crisis in New York, USA [29]). In England, a partnership of community-based organisations have also drawn upon these charters in their development of national HIV prevention planning frameworks, including the current framework, *Making it Count 4*. Past editions of *Making it Count*[30,31] attended to the wider determinants of the behavioural causes of HIV transmission but the current edition [32] is explicit in calling for HIV prevention activity to focus on helping men minimise the harms associated with sex while maximising its benefits. Hickson has simplified this message further to state that HIV health promotion should facilitate men having, “*the best sex with the least harm*” (Hickson, 2011, p.7) [33].

While some community-based organisations (and other health professionals) agree that HIV prevention activity should seek to help men achieve a broader sense of sexual well-being, a detailed consideration and description of what men aspire to for their sex lives does not exist within the published literature. This paper aims to contribute to broader health promotion goals by seeking to understand what MSM value in the context of their sexual lives. We therefore aim to: (1) describe the various dimensions of the ‘best sex’ as articulated by gay and bisexual men, and (2) identify how frequently such dimensions feature in their collective thinking, and (3) describe how men’s ideas of the best sex life vary according to key demographic characteristics.

## Methods

To address these aims we draw on UK data from EMIS [25], a large, multi-national, multi-language survey of sexual behaviour and HIV prevention needs among MSM across Europe. The survey was open for completion between June and August of 2010 and was promoted by a variety of commercial gay social media outlets, including, *Planet Romeo®*, *Manhunt®*, and *Gaydar®* and also by over 200 community websites. There were a total of 174,209 valid responses, representing the largest ever survey of sexual behaviour among men who have sex with men anywhere in the world. A detailed description of the methods has been published elsewhere [34]. EMIS was approved by the Research Ethics Committee of the University of Portsmouth, United Kingdom.

The penultimate, open-ended, question of the survey asked men, “*What’s your idea of the best sex life*?” The exact wording of this question was chosen to encourage men to focus on the best possible sex, rather than just what constitutes good sex, and capture the cognitive, social and subjective realm of sexuality, rather than focusing in on specific sexual acts or behaviours. The focus on ‘best sex *life*’ would, it was hoped, allow men to incorporate contextual factors in their responses that might include longer-term aspirations, rather than single events.

This paper uses data from men living in the UK at the time of survey completion who answered the question, “*What’s your idea of the best sex life*?” in English. A total of 18,435 men living in the UK completed the survey, although to increase data quality we excluded 676 men who gave two or more inconsistent answers across the survey (see Weatherburn et al., 2013 for a description of this procedure), leaving 17,759 men. Of these, 12,942 provided an English language response to the question “*What’s your idea of the best sex life*?” (missing = 4817, of whom 3985 did not answer the question at all and a further 832 gave an answer to this specific question in a language other than English).

Responses to the question ranged from just a few words to several sentences. The mean number of words per response was 11.5. With such a large number of brief responses, we sought to establish a rigorous, but suitably flexible, method of identifying themes and coding responses according to these themes within the data, which drew upon the principles of Framework analysis method [35]. As a starting point, a random sample of 600 responses was selected and reviewed by AB, GH, DR and PW. Each author independently sought to identify and describe all themes they believed to be present prior to a meeting to share findings and start working towards a commonly agreed thematic framework. A first draft of this framework was then used to code another random sample of 600 responses by each of the four authors listed. Conceptual problems with the framework, as well as additional information detail required for each theme was recorded and discussed. This process was repeated with a third random sample of 600 responses to ensure that all necessary dimensions were reflected in the framework, which ultimately comprised eight key themes, plus one additional theme that accommodated responses where the respondent said they were uncertain or not sure.

A fourth sample of 600 responses was then used, with four authors independently coding each response to one or more of the eight key themes. Responses were frequently detailed and multi-dimensional, meaning that two or more themes were often reflected in a single answer. The codes each response had been assigned were then collated and compared to establish inter-rater consistency. Discrepancies were discussed and the focus or description of each theme was adjusted where necessary. This process was repeated a further two times until all were satisfied with the thematic framework, including the conceptualisation of each theme and the way it was described.

This final framework was then utilised by GH to code all 12,942 responses to one *or more* themes. Any ambiguous responses were flagged and further reviewed by AB. Responses which could not be assigned to any theme, or provided insufficient information for coding (n = 813), were recorded as ‘unclassifiable’ and do not feature in the remainder of this analysis (this does not include responses where the respondent explicit said they “*Don’t know*” or were otherwise unsure, which are reflected in the ‘Participant unsure’ theme). In the majority of cases, responses were coded as ‘unclassifiable’ when insufficient information was provided (e.g. “*Having fun*”), where respondents did not answer the question (e.g. “*be happy*”), or where answers were sufficiently ambiguous that they could not be assigned with confidence to any of the eight themes identified (e.g. “*Being honest*” – which might imply being honest with oneself, or with a partner within the confines of a relationship, or being honest with significant others). AB reviewed a random sample of 1000 responses utilising the final framework to ensure consistency of coding with GH. Few inconsistencies emerged and these were resolved. After removal of unclassifiable responses, the final number of responses coded using the thematic framework, and reported on in the remainder of this paper, was 12,129.

Descriptive quantitative analysis was undertaken with SPSS PC version 20. Chi-square and ANOVA were used to establish associations between the citing of themes and a number of sample descriptors: sexual identity; relationship status; the extent to which men were ‘out’ to friends, family or colleagues about their attraction to men (outness); HIV testing history; and age.

## Results

Table 1 provides key demographic information relating to the 12,129 men who were resident in the UK and completed the EMIS survey in English and provided a response to, “*What is your idea of the best sex life?”*, that could be coded to one of the eight themes described later in this section (or were coded as ‘participant unsure’). Figures are rounded to the nearest tenth of a per cent and therefore columns may not up to 100%.

**Table 1:** Key participant demographic information

**Sexual identity**	**n**	**%**	**HIV testing history**	**n**	**%**
Gay/homosexual	10170	84.1	Never (n=3296)	3296	27.3
Bisexual	1322	10.9	Positive (n=1274)	1274	10.6
Other	594	4.9	Negative (n=7505)	7505	62.2
**Relationship with a man/men**	**n**	**%**	**Age (categories)**	**n**	**%**
Not in relationship	7739	64.0	<20 (n=524)	524	4.3
In a relationship	4355	36.0	20s (n=3187)	3187	26.3
**Out to people who know you**	**n**	**%**	30s (n=3120)	3120	25.7
All or almost all	6145	50.9	40s (n=3041)	3041	25.1
More than half	2194	18.2	50+ (n=2257)	2257	18.6
Less than half	1284	10.6	**Age (n=12941)**	**Years**	
Few	1746	14.5	Mean	37.84	
None	694	5.8	SD	12.557	

Our sample, like all opportunistic samples of MSM in England, was predominantly gay identified, well-educated and employed. With a mean age of 37 years, they are somewhat older than samples recruited in gay scene venues (e.g. 32 years in a 2003/4 sample) [36] but similar to other samples recruited online (e.g. 35 years in a 2007/8 sample [37]; 37 years in a 2008 sample [38]). Perhaps because the current sample was predominantly recruited through gay dating sites, the proportion of men currently in a regular relationship was lower (at 36.0%) than in other convenience samples (e.g. 43.8% in a 2007 sample recruited though diverse sources [39]). It is possible that this may have biased the balance of descriptions of the best sex life by over-emphasising the relationships which men do not have (and are looking for).

**Figure 1:**
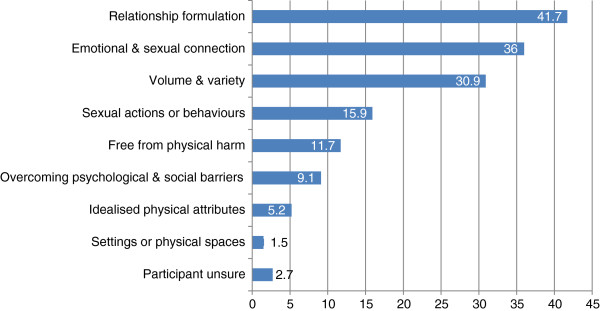
Proportion of responses including each theme (not mutually exclusive).

The eight key themes identified during analysis were: ‘*Emotional and sexual connection*’; ‘*Sex free from physical harm’*; ‘*Overcoming psychological and social barriers’*; ‘*Relationship formulations’*; ‘*Volume and variety of sex’*; ‘*Physical attributes of sexual partner(s)*’; ‘*Sexual actions or behaviours’*; and ‘*Settings or physical spaces for sex’*. These are described in more detail, including indicative responses, in the following section. Responses could be coded to one or more of these themes. For example, the response, “*Safe sex with someone you love*” can be seen to comprise dimensions of ‘Sex free from physical harm’ and ‘emotional or sexual connection’. The themes are described below in the order in which they were most commonly coded. Figure 1 displays the exact percentage occurrence of each theme among UK-based, English language EMIS respondents. Quotes from respondents, shown in *italics*, are provided as theme exemplars.

### Relationship formulations

When asked what their idea of the best sex life was, the most common response (by 41.7% of respondents) described a desire for a relationship with another man (if, indeed, they were not already in one). The extent to which this theme was reflected in responses varied from “*with a boyfriend*”, through to “*A monogamous committed relationship with someone who loves me for who I am”.* Some briefly described, “*With a partner*”, while others detailed their desire for, “*A husband I can be with forever*”. Other responses include: *“The best sex life is the one with only one partner for a number of years in a monogamous relationship”; “Being in love in a committed relationship”; and “With a life partner. It’s not about sex, it’s about making love”.* While monogamous relationships were more commonly cited, or at least strongly inferred in responses that included words such as ‘*committed’*, a sizeable proportion of men also described their best sex life in terms of polygamous or open relationships, sex with their partner and third parties, or other formulations of non-monogamous relationships. For example, “*Regular sex with your boyfriend combined with an open relationship and a date with a sex buddy every week or so*” and “*Have a regular lover with whom you make love regularly and have recreational sex with others from time to time*”.

### Emotional and sexual connection

Over a third (36%) of men stated their desire for some form of loving, intimate or trusting connection with their sexual partner. These responses related to mutuality between themselves and their sexual partner; they generally sought a mutually satisfying and sensual experience. This theme encompasses notions of compatibility, respectfulness and affection. Typical responses include, *“Two people that totally connect with each other on all levels”; “One with a person I’m in love with”; “Sex not just to fulfil desires or lust but to please one another with love, passion and care”.* There was significant overlap in responses that were coded to the theme of ‘*Emotional and sexual connection’* and ‘*Relationship fomulation’*, although the former encompasses feelings and emotions (which form the basis of many relationships) while the latter focuses on technical categorisations of relationships.

### Volume and variety of sex

Nearly a third (30%) of men described their idea of the best sex life in terms of volume of sex or sexual partners, or in the variety of sex they wanted to have. They frequently described wanting sex, “*All the time”*, or “*Frequently with lots of different men”, “As often as I can get it”*, or specified more exact time frames and quantities such as, “*Having sex whenever I want (e.g. more than four times a week mostly on a daily basis)”*. Some men simply stated a desire for any sex, or more than they were currently having. Other responses stated a desire for sex that was varied, adventurous, experimental or exploratory. Typical responses included: “*Total freedom and lots of variation”; “A guy who has similar sexual interests and helps push boundaries and experiences”.* Occasionally men described a range of sexual behaviours which, by their nature and breath, actively implied a desire for variety in their sex life.

### Sexual actions or behaviours

Around one in six men (15.7%) described their idea of the best sex, either solely or in part, in terms of specific sexual acts or behaviours. These include what might be considered the ‘mechanics’ of sex, such as oral sex, anal sex, or masturbation as well as other intimate physical acts between two or more people, such as kissing, massage or frottage (the rubbing of one’s penis against any part of another’s body without penetration). Men often described the physical role they wanted to adopt during sex, such as being the insertive (‘top’) or receptive (‘bottom’) partner in anal intercourse and the manner in which they wished these roles enacted - such as being dominant or submissive during sex. Typical responses include: *“I'm passive so best sex life would be with a good looking 7–8 inch top who can fuck well”*; or “*Being a passive boy for fit aggressive dominant tops”*. This theme captures stated desires for group sexual activity (threesomes etc.) as well as less common sexual practices, such as urination on one or both partners, or fisting (which involves the insertion of one partners’ hand into the other’s anus). For example, *“A wild orgy of men that lasts for hours”; “Fisting and being fisted”.*

### Sex free from physical harm

A relatively small proportion of men (11.7%) described their idea of the best sex in ways that encompassed a desire for sex without physical harm, or the risk of it. Many such responses explicitly stated a desire to avoid HIV or other STIs, such as: ***“****When you can have sex without the worry of STDs”; “Regular unprotected sex with other HIV negative men*”; “*Where there is no HIV or STI's and we can have unprotected safe sex with any willing partner”.* A few men talked more broadly about ‘safe’ or ‘safer’ sex, ‘healthy sex’, or a desire for sex in which they did not feel they would be exposed to HIV. Typical responses include: “*Safe, healthy and lots of it*”; “*Risk free with lots of variety”*; “*Uninhibited sex with whoever, wherever I feel it appropriate to do so, without fear of infection or disease”.* This theme also covers a small number of responses that described the best sex life as consensual and free from coercion. For example, “*One that has the consent of all the people involved”.*

### Overcoming psychological and social barriers

For 9.1% of respondents the best sex life meant being able to have sex without psychological or social barriers, or it meant having the capacity to overcome them. Psychological barriers to the best sex life included a lack of self-confidence (including body-confidence) or assertiveness skills, or a general inability to negotiate the kind of sex desired. Some men described wanting to feel more comfortable and relaxed during sex, or simply to feel less inhibited about actioning their sexual desires. A few described feelings of internalised homonegativity and wanted to feel more comfortable with their sexual orientation and with having sex with men. Typical responses relating to psychological barriers were: “*If I could be more open and relaxed*”; “*Being able to do whatever I want with a consenting partner/partners without fear of being looked down on for doing so*”; “*Being comfortable enough in your own skin to know that who you're with loves you for who you are*”. This theme also encompasses social barriers to the best sex life, including a desire to eliminate or overcome negative attitudes about gay and bisexual men held in some elements of society, and the ability to overcome HIV related stigma. For example, “*Open bisexuality without fear of consequences of disclosure*”; “*Considerate, mutually satisfying & fun and free from the fear of being stigmatised as being HIV positive (not ‘clean’ as is often referred to on Manhunt* [a gay dating website]*)”;* “*When we are all treated the same regardless of HIV status*”.

### Physical attributes of sexual partner(s)

A small number of men (5.2%) associated their idea of the best sex life with sexual partners who had particular physical attributes, such as having a large penis, muscled or toned body, being hairy or smooth or being tall. For example: *“To regularly have sex with a men with a huge penis (>20 cm)”;* “*Loads and often with my sexy muscular man!!”* Some respondents also described a preference for particular ethnicities (e.g. “*Getting fucked by cute Asian man with good size dick”*) or for older or younger sexual partners (e.g. “*Relaxed with good looking young guy*”; “With *two male partners over 60 years old - one fucking me whilst I fuck the other”)*. Physical attributes were also referred to in a more holistic sense by using terminology common-place within gay communities to describe sub-types of gay men, such as ‘bears’ (hirsute men who are usually of a heavier build), ‘cubs’ (smaller versions of ‘bears’), ‘Otters’ (slim, hirsute men), ‘twinks’ (younger, usually quite slim men’), ‘leather guys’ (men who often dress in leather), ‘Skinheads’ (men who shave their heads and often have piercings and/or tattoos), or simply men who are masculine (‘masc’) or feminine (‘fem’) in appearance. Typical responses include: “*Being with a masculine straight acting active top guy”;* or *“Skinhead one-night stands”.* Responses that included general words associated with physical attractiveness (e.g. ‘hot’, ‘cute’, ‘fit’, ’hunky’, ‘good-looking’) were also coded to this theme.

### Settings or physical spaces for sex

For a very small number of men (1.5%), the best sex life was defined by the setting or physical space in which sex occurs. For example, sex in a sauna, a cruising ground, or on a beach. *“On the beach at sunset with the man of my life”; “Outdoor cruising and being watched by other men while having sex”; “A dirty builder’s yard with men ‘pigging’ it in hi-vis gear and dirty jeans and muddy boots”.*

Finally, a few men simply stated that they don’t know or were not sure of their idea of the best sex life.

### Frequency of theme occurrence

Figure 1 displays the frequency with which responses were coded to one or more of those themes described above.

Responses most commonly related to ideal relationships (41.7%), and sexual partnerships that contain some element of emotional or sexual connection (36.0%). Conceptualising best sex in terms of volume or variety of sexual activity was also commonplace (described in some manner by 30.9%). All other themes contained answers from fewer than 20% of respondents.

Some associations were observed between themes. Men whose response related to relationship formulation were also likely to mention some element of emotional and sexual connection (for example, “*Regular sex in a steady, loving relationship*”). Almost two thirds (62.1%) of responses were coded and included in either ‘Relationship formulation’ or ‘emotional and sexual connection’. A total of 15.6% of responses were included in both of these themes.

### Demographic variation

Following coding to one or more themes, responses were compared according to key demographic criteria, as shown in Table 2. Chi-square analysis identified significant differences according to sexual identity in that the responses of gay/homosexual identifying men were more likely to include an element of emotional and sexual connection with a partner, and relationship formulation, than were responses from bisexual identified men. Conversely, bisexual men were more likely than gay/homosexual men to describe their ideal sex life in terms of sexual actions or behaviours.

**Table 2:** Demographic variation in best sex themes represented in each response

		**% of respondents whose answers were included in a theme**								
		**Relationship formulation**	**Emotional & sexual connection with partner**	**Volume & variety**	**Sexual actions or behaviours**	**Free from physical harm**	**Overcoming psychological and social barriers**	**Idealised physical attributes**	**Settings or physical spaces**	**Participant unsure**
**Sexual identity**	Gay/homosexual (n=10170)	**43.7	**37.2	**31.4	**15.2	**11.2	**8.7	5.2	1.6	**2.4
	Bisexual (n=1322)	29.7	27.2	29.5	21.0	14.2	10.8	6.0	1.2	4.3
	Other (n= 594)	33.8	35.7	26.4	16.8	14.0	12.6	4.9	1.0	4.7
**Relationship with a man/men**	Not in relationship (n=7739)	**44.6	**37.6	**28.8	**15.1	**11.1	8.8	5.5	**1.3	**3.0
	In a relationship (n= 4355)	36.6	33.3	34.6	17.6	12.7	9.7	4.7	1.9	2.2
**Out to people who know you**	All or almost all (n=6145)	**42.7	**37.2	**32.6	**16.2	**11.6	9.2	**4.7	1.5	**2.3
	More than half (n=2194)	45.4	38.9	30.5	13.5	10.2	8.3	4.9	1.6	2.6
	Less than half (n=1284)	42.8	36.4	29.9	14.2	10.7	9.7	5.5	1.8	3.0
	Few (n=1746)	38.1	31.5	27.0	18.2	13.2	9.3	7.1	1.3	3.6
	None (n=694)	28.5	26.8	29.5	19.6	14.8	9.9	6.1	1.4	4.8
**HIV testing history**	Never (n=3296)	**38.6	**34.8	31.6	15.7	**10.5	**9.6	**5.6	1.2	**3.8
	Positive (n=1274)	38.4	34.8	29.4	18.3	9.3	10.7	6.8	2.0	3.2
	Negative (n=7505)	43.7	36.8	30.9	15.6	12.6	8.6	4.8	1.6	2.2
**Age**	<20 (n=524)	**39.1	**31.7	**37.8	**12.2	**15.1	8.8	**5.0	**1.0	**3.8
	20s (n=3187)	44.8	38.8	36.9	12.6	12.8	8.9	4.1	0.9	2.3
	30s (n= 3120)	43.3	37.8	31.6	15.2	12.2	8.4	4.6	1.7	2.6
	40s (n=3041)	39.7	33.8	29.1	17.8	11.3	9.2	5.7	1.8	2.6
	50+ (n=2257)	38.5	33.6	22.6	20.1	9.1	10.1	7.1	1.8	3.6

Cross-tabulations shown with ** on the principle line indicate where differences in responses between demographic groups are significant, p < 0.05.

Those in a current relationship with another man were more likely to idealise emotional and sexual connection, and less likely to idealise volume and variety in their sex life than were those not in a relationships. As a general trend, the more out men were to their family, friends and work/study colleagues the more likely they were to idealise emotional and sexual connection. The same was also true of idealising relationship formulation. Those out to few or none were, however, more likely to describe their idea of the best sex life in ways that relate to sexual actions or behaviours.

**Figure 2:**
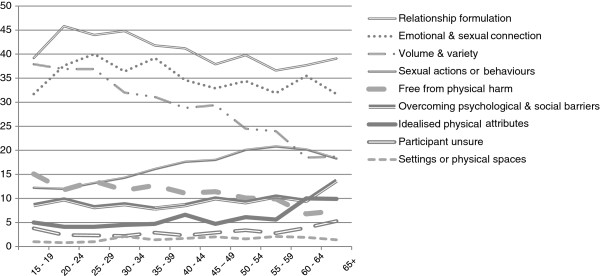
Age variation in best sex themes represented in each response.

A significant difference in idealised sex lives is also evident across age groups. Figure 2 illustrates how the likelihood of describing the best sex life in terms of a relationship formulation, emotional or sexual connection with a partner, or volume and variety with sex all decrease with advancing age. However, the likelihood of describing the best sex life in terms of types, actions or behaviours during sex generally increased in line with age.

The rank order of the eight themes was almost identical across the three testing history groups, suggesting no major group differences in sexual values. However, relationship formulation was significantly more commonly cited by men who had tested negative than those who had never tested or tested positive, as were emotional and sexual connection with a partner, and freedom from physical harm. Conversely, men who had tested HIV positive were significantly (if marginally) more likely to cite overcoming psychological and social barriers and idealised physical attributes than men who had not tested positive. We cannot say whether these differences preceded and perhaps contributed to men staying HIV negative or becoming HIV positive, or whether the differences are a consequence of diagnoses. On the other hand men who had never tested were most likely to be unsure of what their best sex life might contain, perhaps reflecting a broader ambivalence about the world.

## Discussion

This paper describes an exploratory analysis of responses to one open question about what constitutes the best sex life, which were often only several words in length and occasionally ambiguous. A more in-depth, purely qualitative investigation of this issue may reveal greater complexity and richness to these themes, or better illustrate how they are interconnected. As with all social survey research, this data may reflect a social desirability bias, particularly given that common place discourse surrounding the desire for a partner or for love is widely accessible. It is likely that responses are context-dependent, thereby offering a snapshot into a range of men’s ideas of the best sex at one given point in their lives.

Nevertheless, the data we describe paint a detailed and comprehensive picture which contributes to our understanding of what many men hold as the best sex life and the sizeable number of responses helps to reflect the scale of diversity in gay and bisexual men’s thinking on this issue. Comparisons between demographic groups are tentative but provide some useful insight into how ideas of the best sex life, and its role in a broader sense of sexual well-being, may differ among men at different points in their lives or in different personal circumstances. The principle of health promotion conceived by the Ottawa and Bangkok Charters notwithstanding, a significant body of social marketing and communication literature highlights the importance of attending to what people value and what is meaningful in their everyday lives when attempting to elicit health behaviour change [40,41]. Such an understanding on the part of health and social care professionals seeking to reduce transmission of HIV and other STIs among gay and bisexual men may assist in the development of engaging and effective interventions to help men make sexual choices that maximise pleasure or satisfaction and minimise potential harms.

That gay, bisexual and other men who have sex with men value emotional connection or meaningful, romantic relationships with other men is by no means a new finding [42,43] but what the current study adds is better sense of the primacy placed on these themes as key components of an idealised sex life. Nearly two-thirds of respondents described their idea of the best sex life in these terms, while responses relating to variety of sexual contact or specification of sexual acts were substantially less common. This finding stands in stark contrast to a predominate media and commercially driven representation of gay men as promiscuous and interested primarily in anal intercourse [44]. While a still sizeable proportion of men described their idea of the best sex in ways that related to volume and variety, such articulation decreased with the advancing age of respondents. As age increased so too did a tendency to describe specific sexual acts or behaviours, perhaps reflecting how some men gain a greater sense of what is sexually satisfying with experience. Conversely, that relationships featured more strongly in younger men’s responses may reflect maturation effects (whereby younger men have always been more interested in relationships and that this interest declines, or broadens, with age) or changing generational values (with older men who were part of the liberation movement placing less emphasis on relationships and younger men, being more socially integrated, valuing heteronormative relationships). The current study is unable to unpick these maturational and chronological processes (but see Weeks, 2007 [45]).

Public health interventions for HIV prevention emphasise risk reduction but pay little attention to pleasure promotion. This is clearly at odds with what men themselves are aiming for in their sex lives. The purpose of safer sex has always been to continue to have an adequate sexual life in the presence of potential harms by reducing the probability of those harms. The current study suggests that the kind of sex lives MSM aspire to are varied and multifaceted. Interventions should therefore not presuppose what a good sex life is, but instead endeavour to equip men with the skills, awareness and resources to enable them to move towards better sex with less harm.

Some existing community-based HIV prevention interventions have sought to consider the wider sexual health and well-being of MSM, which intersect with several of the themes described in this paper. Particularly common place are online or printed guides to having good sex between men (for examples see ‘The Good Gay Sex Guide’ [46] produced by charity *Gay Men’s Health* in Scotland, and ‘Sex tips for men’ [47] produced by *The Lesbian & Gay Foundation*). Many of these publications seek to help men have enjoyable sex while also highlighting means of avoiding HIV/STI transmission. However, even interventions such as these have a tendency to focus on more mechanical aspects of sex (such as how to effectively perform fellatio or how to feel comfortable during receptive anal intercourse) rather than the more psycho-social dimensions of emotional connection, intimacy and relationships to which men taking part in EMIS clearly aspired. An intervention by the *AIDS Committee of Toronto* and the *Gay Men’s Sexual Health Alliance* in Canada called, ‘*The sex you want’*[48] aims to help men strike a balance between pleasure and safer sex, stressing they need not act in opposition to one another. While it does incorporate information about how to deal with confidence or self-esteem issues as they relate to sex, the extent to which it facilitates a broader sense of well-being and emotional connection with sexual partners is limited. Of course, it is impossible for any single intervention to meet the diverse needs of an equally diverse population of gay and bisexual men.

Psychosocial interventions to help individuals and couples to increase their capacity for intimacy and emotionally connected relationships do exist (see, for example, PACE Health in London, UK [49] and ACON in New South Wales, Australia [50]), however these are often small-scale and lack the visibility of mass-media interventions, which tend to focus on HIV testing options and condom use (e.g. the ‘Testing makes us stronger’ [51] intervention by the *Centres for Disease Control* in the USA and the forthcoming ‘HIV testing week’ [52] intervention, coordinated by *HIV in Europe*). A larger scale and innovative intervention of particular note [53] is that of the *Victoria AIDS Council* in Australia, which presented information about sexual health and well-being through the medium of social networking based ‘webisodes’: short films in a narrative ‘soap opera’ format. This approach facilitated the presentation and discussion of gay relationships, including associated thoughts and feelings of the characters, as well as articulating information about how to have safer sex that limits the possibility of HIV or other STI transmission. The data we have described serves to emphasise the diversity and plurality of men’s sexual aspirations, which extend far beyond a desire to avoid HIV or STI transmission. Further creative or innovative ways to help men achieve a broader sense of sexual well-being need to be identified, up-scaled and resourced.

The emergence of new HIV prevention options that utilise anti-retroviral therapy reinforces the need for early diagnoses of HIV and the urgency of increasing HIV testing among those sexually active in higher risk groups. However, with an ever increasing focus on prevention of infection by testing and medical treatment, it is possible that HIV prevention activity may move further still from the principles of health promotion that seek to achieve more than the absence of disease or infection. Numerous authors have highlighted the importance of maintaining a focus on the psychological and socio-cultural dimensions of sexual health in the move towards ‘test and treat’ models of HIV prevention [54-56]. Emerging medical technologies may have the potential to prevent many new infections, but these will only be successful if they take account of the lived experience and beliefs (as well as community and broader social structures) of individuals most at risk of contracting HIV, including what they value most in the context of their sexual lives. Interventions that utilise new medical HIV prevention technologies, as well as those that rely on condom use, need to take better account of the themes presented above to ensure that their translation from controlled trial to wider population is as efficacious as possible. In particular, there is clear potential to link oral chemoprophylaxis with antiretrovirals or ‘Treatment as Prevention’ interventions among MSM in sero-discordant relationships with desires for emotional or sexual connection (particularly as many strive towards greater intimacy within their sexual relationships).

Future studies may wish to use the broad themes described in this paper as the basis of further quantitative enquiry, which may enable more sophisticated analysis of how constructions of the best sex life differ according to demographic groups, relationship status or HIV status, and how they correlate with current or recent feelings of sexual happiness. A quantitative study informed by these findings may also allow for a detailed analysis of how these themes overlap in men’s thinking as they conceptualise their idea of the best sex life. The themes may also assist in the future development of a scale to assess sexual aspirations or sexual satisfaction among gay and bisexual men, or in the development of more in-depth qualitative research that explores sexual well-being among this population. The findings described in this paper reflect the views of men resident in the United Kingdom who were completing the survey in English. There is a need to explore if and how constructions of the best sex differ among men from different social, cultural, national or linguistic backgrounds.

## Conclusions

The notion of the ‘best sex life’ is complex, subjective and highly contingent upon individual circumstance and life situation. Data described in this survey are diverse but cluster around several key themes which highlight the primacy of meaningful interaction with other men as a key component of the best sex life. As HIV prevention activity becomes increasingly medicalised, with an associated focus on HIV testing and pharmaceuticals, it is important that the broader sexual well-being of gay and bisexual men is not forgotten. Sexual health does not begin and end with HIV and the extent of sexual unhappiness previously documented among this population should be a cause for concern for all those working across the clinical, health, human rights and social care spectrum. By taking account of what men value or aspire to in the context of their sexual lives we may be able to develop interventions that are both engaging and valuable for them in achieving the best possible sex, while at the same time facilitating an environment in which HIV transmission may be less likely to occur.

## Competing interests

The authors declare that they have no competing interests.

## Authors’ contributions

The survey was designed and executed by AJS, FH, DR and PW in association with the EMIS Network (see Acknowledgements). FH conceived the question relating to the ‘best sex life’. The thematic framework was devised by AB, GH, DR and PW. Coding of the data was performed by GH, supported by AB. Demographic comparative analysis was conducted by FH & DR. The manuscript was drafted by AB and commented on by all other authors. AB and FH prepared the revision. All authors approved the final manuscript.

## Pre-publication history

The pre-publication history for this paper can be accessed here:

http://www.biomedcentral.com/1471-2458/13/1083/prepub

## References

[B1] Health Protection Agency HIV in the United Kingdom: 2012 report 2012 Collindale, London: Health Protection Agency

[B2] Baral S Sifakis F Cleghorn F Beyrer C Elevated risk for HIV infection among men who have sex with men in low- and middle-income countries 2000–2006: a systematic review PLoS Med 2007 4 12 e339 10.1371/journal.pmed.0040339 18052602 PMC2100144

[B3] Sullivan PS Hamouda O Delpech V Geduld JE Prejean J Semaille C Kaldor J Folch C Op de Coul E Marcus U Reemergence of the HIV epidemic among men who have sex with men in North America, Western Europe, and Australia, 1996–2005 Ann Epidemio 2009 19 6 423 431 10.1016/j.annepidem.2009.03.004 19460672

[B4] Bourne A Dodds C Keogh P Weatherburn P Hammond G Relative safety II: risk and unprotected anal intercourse among gay men with diagnosed HIV 2009 London: Sigma Research

[B5] Crepaz N Marks G Liau A Mullins MM Aupont LW Marshall KJ Jacobs ED Wolitski RJ Prevalence of unprotected anal intercourse among HIV-diagnosed MSM in the United States: a meta-analysis AIDS 2009 23 13 1617 1629 10.1097/QAD.0b013e32832effae 19584704

[B6] Rawstorne P Fogarty A Crawford J Prestage G Grierson J Grulich A Kippax S Differences between HIV-positive gay men who ‘frequently’, ‘sometimes’ or ‘never’ engage in unprotected anal intercourse with serononconcordant casual partners: positive Health cohort, Australia AIDS Care 2007 19 4 514 522 10.1080/09540120701214961 17453592

[B7] Ross MW Berg RC Schmidt AJ Hospers HJ Breveglieri M Furegato M Weatherburn P Internalised homonegativity predicts HIV-associated risk behavior in European men who have sex with men in a 38-country cross-sectional study: some public health implications of homophobia BMJ Open 2013 3 2 392 396 doi:10.1136/bmjopen-2012-00192 10.1136/bmjopen-2012-001928 PMC3586183 23386580

[B8] Ruf M Lovitt C Imrie J Recreational drug use and sexual risk practice among men who have sex with men in the United Kingdom Sex Transm Infect 2006 82 2 95 97 10.1136/sti.2005.018317 16581728 PMC2564699

[B9] Volk JE Prestage G Jin F Kaldor J Ellard J Kippax S Grulich AE Risk factors for HIV seroconversion in homosexual men in Australia Sex Health 2006 3 1 45 51 10.1071/SH05020 16607974

[B10] Zablotska I Imrie J Prestage G Crawford J Rawstorne P Grulich A Jin F Kippax S Gay men’s current practice of HIV seroconcordant unprotected anal intercourse: serosorting or seroguessing AIDS Care 2009 21 4 501 510 10.1080/09540120802270292 19266409

[B11] Rouwenhorst E Mallitt KA Prestage G Gay men’s use of condoms with casual partners depends on the extent of their prior acquaintance AIDS Behav 2012 16 6 1589 1596 10.1007/s10461-011-0092-y 22127551 PMC3401304

[B12] Holt M Mao L Prestage G Zablotska I De Wit J Gay community periodic surveys: national report 2010 2011 Sydney: National Centre in HIV Social Research, The University of New South Wales

[B13] Weatherburn P Hickson F Reid D Jessup K Hammond G Multiple chances: findings from the United Kingdom gay men’s sex survey 2006 2008 London: Sigma Research

[B14] Cohen MS Chen YQ McCauley M Gamble T Hosseinipour MC Kumarasamy N Hakim JG Kumwenda J Grinsztejn B Pilotto JH Prevention of HIV-1 infection with early antiretroviral therapy N Engl J Med 2011 365 6 493 505 10.1056/NEJMoa1105243 21767103 PMC3200068

[B15] Grant RM Lama JR Anderson PL McMahan V Liu AY Vargas L Goicochea P Casapia M Guanira-Carranza JV Ramirez-Cardich ME Preexposure chemoprophylaxis for HIV prevention in men who have sex with men N Engl J Med 2010 363 27 2587 2599 10.1056/NEJMoa1011205 21091279 PMC3079639

[B16] Prestage G Mao L Kippax S Jin F Hurley M Grulich A Imrie J Kaldor J Zablotska I Use of viral load to negotiate condom use among gay men in Sydney, Australia AIDS Behav 2009 13 4 645 651 10.1007/s10461-009-9527-0 19199021

[B17] Young I Li J McDaid L Awareness and willingness to use HIV pre-exposure prophylaxis amongst gay and bisexual men in Scotland: implications for biomedical HIV prevention PLoS One 2013 8 5 e64038 10.1371/journal.pone.0064038 23691143 PMC3656929

[B18] Mao L Newman CE Kidd MR Saltman DC Rogers GD Kippax SC Self-reported sexual difficulties and their association with depression and other factors among gay men attending high HIV-caseload general practices in Australia J Sex Med 2009 6 5 1378 1385 10.1111/j.1743-6109.2008.01160.x 19170866

[B19] Sandfort TG de Keizer M Sexual problems in gay men: an overview of empirical research Annu Rev Sex Res 2001 12 93 120 12666738

[B20] Pawlicki P Larson P The dynamics and conceptualizations of non-exclusive relationships in gay male couples Sexual and Relationship Therapy 2011 26 1 48 60 10.1080/14681994.2010.516247

[B21] LaSala MC Extradyadic sex and gay male couples: comparing monogamous and nonmonogamous relationships Families in Society: The Journal of Contemporary Human Services 2004 85 405 412 10.1606/1044-3894.1502

[B22] Carballo-Dieguez A Ventuneac A Dowsett GW Balan I Bauermeister J Remien RH Dolezal C Giguere R Mabragana M Sexual pleasure and intimacy among men who engage in “bareback sex” AIDS Behav 2011 15 1 S57 S65 21380496 10.1007/s10461-011-9900-7 PMC3319084

[B23] Prestage G Down IA Bradley J McCann PD Brown G Jin F Hurley M Is optimism enough? Gay men’s beliefs about HIV and their perspectives on risk and pleasure Sex Transm Dis 2012 39 3 167 172 10.1097/OLQ.0b013e31823e67a9 22337101

[B24] Bourne A Hickson F Keogh P Reid D Weatherburn P Problems with sex among gay and bisexual men with diagnosed HIV in the United Kingdom BMC Public Health 2012 12 916 10.1186/1471-2458-12-916 23107161 PMC3503855

[B25] The EMIS Network The european men-who-have-sex-with-men internet survey. Findings from 38 countries 2013 Stockholm: European Centre for Disease Prevention and Control

[B26] World Health Organisation Ottawa charter for health promotion First international conference on health promotion. 21 november 1986; Ottawa, Canada 1986

[B27] World Health Organisation The bangkok charter for health promotion in a globalized world6th global conference on health promotion. august 2005 2005; Bangkok, Thailand 2005

[B28] ACON, revolutions: ACON strategic plan 2009–2012 http://www.acon.org.au/sites/default/files/ACON-Strategy-2009-Final.pdf

[B29] Gay men’s health crisis: About ushttp://www.gmhc.org/about-us

[B30] Hickson F Nutland W Weatherburn P Burnell C Keogh M Doyle T Watson R Gault A Making it count: a collaborative planning framework to reduce the incidence of HIV infection during sex between men 2003 3 London: Sigma Research

[B31] Hickson F Nutland W Doyle T Burbidge N Burnell C Cadette M Henderson L Ward M Woolls C Making it count: a collaborative planning framework to reduce the incidence of HIV infection during sex between men (2nd edition) 2000 London: Sigma Research

[B32] Partnership CHAPS Making it count 4: a collobaroative framework to minimise the incidence of HIV infection during sex between men (4th edition) 2011 London: Sigma Research

[B33] Hickson F Towards better sex with less harm for gay and bisexual men in Europe Oral presentation at: *FEMP: the future of HIV prevention among MSM in europe: 9th november 2011; Stockholmc Sweden* 2011 London: Sigma Research http://www.sigmaresearch.org.uk/files/HIckson_FEMP_Plenary_Better_sex_less_harm.pdf

[B34] Weatherburn P Schmidt AJ Hickson F Reid D Berg RC Hospers HJ Marcus U The european men-who-have-sex-with-men internet survey: design and methods Sex Res Soc Policy 2013 doi:10.1007/s13178-013-0119-4

[B35] Ritchie J Spencer L Bryman A, Burgess R Qualitative data analysis for applied policy research Analyzing qualitative data 1994 London: Sage 173 194

[B36] Dodds JP Johnson AM Parry JV Mercey DE A tale of three cities: persisting high HIV prevalence, risk behaviour and undiagnosed infection in community samples of men who have sex with men Sex Transm Infect 2007 83 5 392 396 10.1136/sti.2006.021782 17472978 PMC2659037

[B37] Elford J Doerner R McKeown E Nelson S Anderson J Low: HIV infection among ethnic minority and migrant men who have sex with men in Britain Sex Transm Dis 2012 39 9 678 686 10.1097/OLQ.0b013e31825c8018 22902663

[B38] Hickson F Bonell C Hargreaves J Reid D Weatherburn P HIV testing and HIV serostatus-specific sexual risk behaviour among men who have sex with men living in England and recruited through the internet in 2001 and 2008 Sex Res Soc Policy 2013 10 1 15 23 10.1007/s13178-012-0106-1 PMC4557419 26361522

[B39] Hickson F Weatherburn P Ried D Jessup K Hammond G Testing targets: findings from the united kingdom gay men’s sex survey 2007 2008 London: Sigma Research http://www.sigmaresearch.org.uk/files/report2009f.pdf

[B40] Grier S Bryant CA Social marketing in public health Annu Rev Public Health 2005 26 319 339 10.1146/annurev.publhealth.26.021304.144610 15760292

[B41] Lamptey PR Price JE Social marketing sexually transmitted disease and HIV prevention: a consumer-centered approach to achieving behaviour change AIDS 1998 12 2 S1 S9 9792356

[B42] Dowsett GW Practicing desire: homosexual sex in the era of AIDS Stanford: Stanford university press 1996

[B43] Rhodes T Cusick L Love and intimacy in relationship risk management: HIV positive people and their sexual partners Sociol Health Illn 2000 22 1 1 26 10.1111/1467-9566.00189

[B44] Mowlabocus S Paasonen C, Nikuenene K, Saarenmaa A Gay men and the pornification of everyday life Pornification 2007 Oxford: Berg

[B45] Weeks J The world we have won 2007 London: Routledge

[B46] The good gay sex guidehttp://gmh.org.uk/sex/introduction.html

[B47] Sex tips for men http://www.lgf.org.uk/get-support/Campaigns/Sex-tips-for-men/

[B48] The sex you wanthttp://www.thesexyouwant.ca/

[B49] PACE groups for menhttp://www.pacehealth.org.uk/services/

[B50] Building our community’s health and well-being: Looking for Mr Righthttp://www.acon.org.au/mens-health/groups-and-workshops/mr-right

[B51] Testing makes us strongerhttp://hivtest.cdc.gov/stronger/

[B52] European HIV testing week 2013 http://www.hiveurope.eu/OngoingProjects/EuropeanHIVTestingWeek2013/tabid/191/Default.aspx

[B53] Pedrana A Hellard M Gold J Ata N Chang S Howard S Asselin J Ilic O Batrouney C F**k SM:Qa reaching and engaging gay men in sexual health promotion through social networking sites J Med Internet Res 2013 15 2 doi:10.2196/jmir.2334 10.2196/jmir.2334 PMC3636214 23391459

[B54] Kippax S Effective HIV prevention: the indispensible role of social science J Int AIDS Soc 2012 15 2 http://dx.doi.org/10.7448/IAS.15.2.17357 10.7448/IAS.15.2.17357 PMC3499803 22713254

[B55] Nguyen VK Remedicalizing an epidemic: from HIV treatment as prevention to HIV treatment is prevention AIDS 2011 25 291 293 10.1097/QAD.0b013e3283402c3e 20962615

[B56] Persson A Non/infectious corporealities: tensions in the biomedical era of ‘HIV normalisation’ Sociol Health Illn 2012 35 7 1065 1079 23278343 10.1111/1467-9566.12023

